# Influence of sex and systemic lupus erythematosus disease status on cerebral cortex structure

**DOI:** 10.1136/lupus-2026-002032

**Published:** 2026-06-25

**Authors:** Yifan Yang, Li Tao, Ruotong Zhao, Ru Bai, Shuang Liu, Guofang Zhang, Shu Li, Xinyu Xu, Yuqi Cheng, Jian Xu

**Affiliations:** 1 Department of Rheumatology and Immunology, First Affiliated Hospital of Kunming Medical University, Kunming, China; 2 Department of Rheumatology and Nephrology, Mile People’s Hospital, Mile, China; 3 Affiliated Mental Health Center, Zhejiang University School of Medicine, Hangzhou, China

**Keywords:** Lupus Erythematosus, Systemic, Qualitative research, Magnetic Resonance Imaging

## Abstract

**Objective:**

To investigate the main and interactive effects of sex and disease diagnosis on cortical morphology in systemic lupus erythematosus (SLE), providing neuroimaging evidence for sex-by-disease interactive mechanisms underlying structural brain alterations.

**Methods:**

A cross-sectional study design was used to recruit participants. Structural MRI scans were acquired and processed using surface-based morphometry (SBM) to extract cortical thickness (CT), gyrification index (GI), fractal dimension (FD) and sulcal depth (SD) within predefined regions of interest. Two-way analysis of variance assessed main effects and ‘sex×diagnosis’ interactions (Holm-Bonferroni corrected). Correlation analyses were performed between these structural metrics and SLE disease activity/psychological assessment scores in areas showing significant interactions.

**Results:**

196 individuals participated in this study: 30 male SLE patients, 66 female SLE patients, 33 male healthy controls (HC) and 67 female HC. Significant sex-by-disease interactions were detected: SD in left orbital (H-shaped) sulci (S_orbital-H_Shaped) (*F*=14.8458, p=0.0150), with male HC >male SLE (p=0.0039) and male HC>female HC (p<0.0001), and GI in left middle frontal sulcus (S_front_middle) (*F*=14.1650, p=0.0313), with female SLE>male SLE (p<0.0001) and male HC>male SLE (p=0.0003). Diagnostic main effects (HC>SLE) were observed extensively for CT across both hemispheres. No significant correlations emerged between disease activity/psychological assessment scores and structural parameters in interaction-significant regions.

**Conclusion:**

This study provides novel evidence for sex-by-diagnosis interactive effects in the neuropathological mechanisms of SLE. Sex and SLE disease status have both independent and interacting impacts on cerebral cortical structure.

**Trial registration number:**

NCT00703742.

**WHAT IS ALREADY KNOWN ON THIS TOPIC**

- Patients with systemic lupus erythematosus (SLE) exhibit widespread cortical structural abnormalities, and sex significantly influences disease epidemiology and severity; however, whether sex and disease status interact to shape cortical complexity remains unknown.

**WHAT THIS STUDY ADDS**

- This study provides the first evidence that sex and SLE diagnosis interact to alter cortical complexity, with male patients showing disproportionate reductions in orbitofrontal sulcal depth and middle frontal gyrification index relative to both male healthy controls and female patients.

**HOW THIS STUDY MIGHT AFFECT RESEARCH, PRACTICE OR POLICY**

- These findings support sex-stratified neuroimaging analyses in SLE to capture male-specific structural vulnerabilities and suggest that sex-tailored screening for early neuropsychiatric risk may be warranted in clinical practice.

## Background

 Systemic lupus erythematosus (SLE) is an autoimmune-mediated connective tissue disorder. Anxiety and mood disorders have been demonstrated to be more prevalent in SLE patients than in the general population.^1^ Notably, significant sex differences exist in the epidemiology and clinical characteristics of SLE. Although the incidence and prevalence of SLE are higher in females than in males,^2^ the incidence of anxiety symptoms among female SLE patients (38.9%) is higher than that among male patients (16.7%).^3^ However, male patients often exhibit higher disease activity,^4^ are more prone to organ damage^5^ and have a significantly higher mortality rate than females. The 5-year survival rate for male SLE patients is 92.0%, compared with 97.6% for females.^6^

From a neuroimaging perspective, SLE patients exhibit structural brain abnormalities, including reduced overall brain volume, particularly marked decreases in hippocampal, corpus callosum and total grey matter volumes.^7^ Compared with healthy individuals, patients with neuropsychiatric SLE (NPSLE) exhibit markedly thinner cortical thickness (CT) in the right insular cortex.^8^ Extensive and pronounced CT reduction has even been observed in patients with non-NPSLE.^9^ Analysis of sex differences indicates that female SLE patients exhibit significantly reduced grey matter volume (GMV) compared with male patients.^10^

MRI is a non-invasive neuroimaging technique that has become a vital tool for investigating structural and functional abnormalities in the brains of SLE patients. Surface-based morphometry (SBM), as an advanced neuroimaging analysis technique, has been employed in several studies to reveal abnormal CT alterations in patients with SLE.^11 12^

In summary, existing evidence suggests that sex may interact with SLE disease status to influence brain structural remodelling. However, research on the interaction between sex and SLE disease on brain structure remains scarce. This study employed the SBM method to investigate the main effects and interactions between sex and SLE disease status in brain structural abnormalities. It aims to provide neuroimaging evidence for the differences in clinical manifestations between male and female SLE patients.

## Methods

### Research participants

The case group consisted of SLE patients who attended the Department of Rheumatology and Immunology of the First Affiliated Hospital of Kunming Medical University, while healthy volunteers were also recruited as healthy controls (HC). In the research process, including the collection of clinical data and acquisition of MRI data, it was conducted using a standardised protocol and was monitored by the same investigator throughout the study. Each study participant underwent interviews with both an experienced rheumatologist and a psychiatrist, along with a comprehensive physical examination focusing on neuropsychiatric manifestations. Inclusion and exclusion criteria were as follows:

SLE group inclusion criteria: (1) Patients meeting the 1997 American College of Rheumatology classification criteria for SLE, with no history or current presence of severe or overt neuropsychiatric disease (mild anxiety, depression or subclinical cognitive deficits were not excluded), and normal cranial MRI T1- and T2-weighted imaging scans (ie, absence of gross structural abnormalities). (2) Age 18 years or older, identified as right-handed according to the Edinburgh Handedness Inventory. (3) Patients who were untreated prior to MRI examination or had received glucocorticoid therapy (at a stable dose for ≥2 weeks), hydroxychloroquine or immunosuppressive agents (eg, mycophenolate mofetil, tacrolimus, cyclosporine, azathioprine, methotrexate, leflunomide) for at least 8 weeks, with the stable dose maintained for at least 4 weeks. (4) Voluntarily participating in this study, having read and signed the informed consent form and able to cooperate with MRI examinations and questionnaire assessments. (5) Female patients who are neither pregnant nor breastfeeding.

Exclusion criteria for the SLE group: (1) Patients with organic brain disorders or neurological conditions affecting brain structure (eg, history of head trauma or surgery, Parkinson’s disease, epilepsy, stroke, etc). (2) History of severe psychiatric disorders such as depression, anxiety disorders, schizophrenia, etc. (3) History of alcohol abuse, substance misuse or prior treatment with antidepressants or antipsychotics. (4) Concurrent autoimmune disorders. (5) Patients with severe clinical manifestations potentially affecting brain structure, such as severe hypertension, diabetes mellitus or renal insufficiency. (6) Receipt of any of the following treatments within the specified pre-enrolment timeframe: plasmapheresis <6 months; rituximab, obinutuzumab, oreguzumab or other B-cell depletion therapies<1 year; belimumab, anifrolumab, abatacept, telitacicept, any anti-TNF (tumour necrosis factor) therapy or other biologics with immunosuppressive potential or likely to interfere with SLE disease activity assessment <3 months; intravenous immunoglobulin or granulocyte colony-stimulating factor < 1 month; cyclophosphamide <1 month; lenalidomide or thalidomide <1 month; strong opioids <1 week; any investigational drug or drug not listed above <5 half-lives. (7) Patients with contraindications to MRI scanning, such as claustrophobia or metallic foreign bodies (eg, dental implants). (8) Patients unable to complete questionnaire assessments or form completion.

HC group inclusion criteria: (1) Matched with the patient group in terms of age, sex and educational attainment. (2) Determined to be right-handed according to the Edinburgh Handedness Inventory. (3) No history of severe physical or mental illness, physically and mentally healthy. (4) Normal conventional cranial MRI T1 and T2-weighted imaging scans. (5) Comprehensive physical examination by an experienced rheumatology and immunology specialist confirming no abnormalities; concurrently, neurological examination by an experienced psychiatrist and screening using the Structured Clinical Interview for Non-Psychotic Disorders based on the Diagnostic and Statistical Manual of Mental Disorders, Fourth Edition, both yielding normal results. (6) Non-lactating or non-pregnant women. (7) Study participants voluntarily consent to this research by signing an informed consent form and are able to cooperate with MRI examinations and questionnaire assessments.

Prior to entry into the study, each participant provided written informed consent after receiving a complete description of the study. This research was approved by the Ethics Committee of the First Affiliated Hospital of Kunming Medical University, Yunnan Province, China (ClinicalTrials.gov, NCT00703742; chictr.org.cn, ChiCTR2200056189).

### Data collection

Data were collected on participants’ age, educational attainment, disease duration and disease activity (systemic lupus erythematosus disease activity index (SLEDAI)). Disease duration was defined as the time from the initial manifestation of SLE to the date of MRI acquisition. Laboratory investigations included complete blood count, blood biochemistry, autoantibody profile, immunoglobulins, complement levels, etc.

### Psychological assessment scales

On the day of the MRI examination, the same psychiatrist administered the following assessments: the Mini-Mental State Examination (MMSE), the Hamilton Depression Scale (HAMD), the Hamilton Anxiety Scale (HAMA), Beck Depression Inventory (BDI) and Self-Rating Anxiety Scale (SAS), to characterise each participant’s intelligence, cognitive function, anxiety and depression for descriptive purposes rather than as exclusion criteria. Disease activity scoring and psychological assessments were conducted within 2 days before and after the MRI examination.

### MRI image acquisition and data processing

#### MRI image acquisition

All MRI images were acquired for study participants by one experienced neuroradiologist using a GE 1.5 T head coil MRI scanner. T1-weighted and T2-weighted imaging sequences were performed initially to exclude gross structural abnormalities. Three-dimensional T1-weighted fast phase-shift gradient echo sequences (3D-T1-fspgr sequences) were employed for 3D structural MRI with the following scan parameters: repetition time (TR)=10.5 ms, echo time (TE)=2 ms, slice thickness=1.8 mm, inter-slice gap=0, flip time=350 ms, scan matrix=256 × 256, flip angle (FA)=15°, number of slices=172 and spatial resolution=0.94 mm × 0.94 mm × 0.9 mm, with the scan range covering the entire brain.

#### MRI data processing

Data preprocessing comprised the following steps: (1) on the MATLAB R2019b (Matswalk Inc., Sherborn, MA, USA) platform, using SPM12 (v7771) (Wellcome Trust Centre for Neuroimaging) and Computational Anatomy Toolbox 12 (CAT12, Structural Brain Atlas Group, University of Jena, Germany) (r2043) to process and analyse T1-weighted MRI images from all study participants. The default mode of the ‘Segment Data’ module in CAT12 was employed: converting all 3D T1WI images into NIfTI format. SBM analysis comprised image segmentation, cortical reconstruction, topological correction, spherical registration and spatial normalisation using the DARTEL algorithm, yielding the central surfaces of the left and right cerebral hemispheres. (2) Quality checks were performed on acquired images, including visual inspection and SPM data quality checks. (3) Structural metrics for the left and right cerebral hemispheres were extracted using the ‘Surface tool’ function in CAT12, including CT, gyrification index (GI), fractal dimension (FD) and sulcus depth (SD). (4) Image smoothing was performed using a 20 mm Gaussian kernel for GI, FD and SD and a 15 mm Gaussian kernel for CT. All procedures adhered to the guidelines provided in the CAT 12 software manual.

#### Patient and public involvement

Patients or the public were not involved in the design, conduct, reporting or dissemination plans of our research.

#### Statistical analysis

Statistical analysis of demographic and clinical data was conducted using SPSS V.25.0. For normally distributed quantitative data, results are presented as mean±standard deviation ($\overset{-}{x}\pm s$). Comparisons between two groups employed the *t*-test, while comparisons among multiple groups used analysis of variance (ANOVA). For non-normally distributed quantitative data, median M (*P*25, *P*75) was used. Comparisons between two groups employed the Mann-Whitney U test, while multiple comparisons used the Kruskal-Wallis rank-sum test with Bonferroni correction. Categorical data were presented as frequencies and rates (%). Comparisons between groups were performed using the χ2 test. A two-tailed *p* value <0.05 was considered statistically significant.

We employed a region-of-interest (ROI)-based SBM analysis method, utilising the a2009s atlas to subdivide each cerebral hemisphere into 74 distinct ROIs. These regions encompass information pertaining to human cerebral gyri and sulci structures.^13^ Two-factor ANOVA was performed using CAT12/SPM12 on smoothed SD, FD, GI and CT indices, with age and years of education as covariates. We explored the main effects of sex (male and female), disease status (SLE and HC) and their interaction on grey matter structure. Multiple comparisons were corrected using the Holm-Bonferroni method to control Type I errors (p<0.05). The CAT toolbox extracted mean structural data from brain regions showing statistically significant intergroup differences. Simple effects analyses were performed on structural data from regions with interaction effects using GraphPad software, with results corrected by Bonferroni (p<0.05/4=0.013). Finally, we conducted Pearson or Spearman correlation analyses between the structural data of brain regions with interaction effects in SLE patients and their SLEDAI, HAMA, HAMD, MMSE, BDI and SAS scores (p<0.05 was considered statistically significant).

## Results

### Analysis of demographic and clinical characteristics of study participants

This study ultimately enrolled 196 participants, comprising 30 males in the SLE group, 66 females in the SLE group, 33 males in the HC group and 67 females in the HC group. There were no statistically significant differences in age or years of education among the four participant groups (p>0.05). ompared with the female SLE group, the male SLE group had higher frequencies of anti-Sm antibody positivity (73.9% vs 44.3%, *χ*² = 5.887, p=0.015) and anti-U1RNP antibody positivity (57.1% vs 28.3%, *χ*² = 5.143, p=0.023). (table 1)

**Table 1:** Statistical results of demographic and clinical data of study participants

	SLE-male (n=30)	SLE-female (n=66)	HC-male (n=33)	HC-female (n=67)	*Z/χ^2^/H*	P value
Age	29.50 (24.50, 38.25)	28.00 (23.00, 36.00)	32.30±6.96	30 (26.00, 37.00)	3.900*	0.273
Years of education	12.00 (9.00, 16.00)	12.00 (9.00, 16.00)	15.00 (12.00, 16.00)	15.00 (12.00, 16.00)	7.639*	0.054
Disease duration (months）	4.00 (1.00, 12.00）	6.00 (2.00, 25.75）	N/A	N/A	−0.859†	0.390
HAMD	8.35±5.44 (n=20)	8.50 (5.75, 14.00) (n=38)	0.00 (0.00, 0.00)	0.00 (0.00, 0.00)	−0.844†	0.399
HAMA	7.05±4.74 (n=20)	7.00 (4.00, 12.25) (n=38)	0.00 (0.00, 0.00)	0.00 (0.00, 0.00)	−0.624†	0.532
MMSE	28.00 (27.00, 29.00) (n=20)	28.00 (23.00, 30.00) (n=32)	30.00 (30.00, 30.00)	30.00 (30.00, 30.00)	−0.092†	0.927
BDI	12.72±6.10 (n=18)	14.61±12.24 (n=34)	N/A	N/A	−0.048†	0.962
SAS	36.38±7.64 (n=18)	37.00 (31.00, 43.25) (n=36)	N/A	N/A	−0.110†	0.912
SLEDAI	13.83±7.91 (n=30)	7.00 (10.00, 16.25) (n=66)	N/A	N/A	−1.243†	0.214
Hypocomplementaemia (%)	22/23 (95.7)	47/61 (77.0)	N/A	N/A	2.774‡	0.096
Anti-dsDNA antibodies (%)	15/22 (68.2)	40/59 (67.8)	N/A	N/A	0.001‡	0.974
Anti-Sm antibodies (%)	17/23 (73.9)	27/61 (44.3)	N/A	N/A	5.887‡	0.015§
Anti-P0 antibodies (%)	11/23 (47.8)	20/61 (32.8)	N/A	N/A	1.622‡	0.203
aCL antibodies Ig(%)	2/3 (66.7)	3/12 (25.0)	N/A	N/A		0.242¶
aCL antibodies IgG (%)	3/20 (15.0)	9/41 (22.0)	N/A	N/A	0.089‡	0.766
aCL antibodies IgM (%)	5/20 (25.0)	11/41 (26.8)	N/A	N/A	0.023‡	0.879
Lupus anticoagulant (%)	1/7 (14.3)	5/22 (22.7)	N/A	N/A	/	1.000¶
Anti-β2-Glycoprotein I antibodies (%)	1/7 (14.3)	5/23 (21.7)	N/A	N/A	/	1.000¶
Anti-phosphatidylserine antibodies (%)	1/7 (14.3)	4/22 (18.2)	N/A	N/A	/	1.000¶
Anti-U1RNP antibodies (%)	12/21 (57.1)	13/46 (28.3)	N/A	N/A	5.142‡	0.023§
Anti-SSA52KD antibodies (%)	9/23 (39.1)	30/61 (49.2)	N/A	N/A	0.678‡	0.410
Anti-SSA60KD antibodies (%)	12/23 (52.2)	38/61 (62.3)	N/A	N/A	0.710‡	0.399
Anti-SSB antibodies (%)	7/23 (30.4)	21/61 (34.4)	N/A	N/A	0.120‡	0.729
Anti-histones antibodies (%)	14/23 (60.9)	25/61 (41.0)	N/A	N/A	2.656‡	0.103
Anti-nucleosome antibodies (%)	12/23 (52.2)	22/61 (36.1)	N/A	N/A	1.799‡	0.180
Anti-centromere antibodies (%)	1/23 (4.3)	1/60 (1.7)	N/A	N/A	0.508‡	0.476
Anti-DNP antibodies (%)	4/17 (23.5)	4/30 (13.3)	N/A	N/A	0.240‡	0.624

* SLE-male, SLE-female, HC-male, and HC-female, Kruskal-Wallis rank sum test, with the test statistic being *H*.

† SLE-male vs SLE-female, Mann-Whitney U test, with the test statistic being *Z*.

‡ SLE-male vs SLE-female, χ2 test, with the test statistic being *χ*2.

§ *P* < 0.05, indicating a statistically significant difference.

¶ SLE-male vs SLE-female, Fisher’s exact probability test.

BDI, Beck Depression Inventory; CTX, cyclophosphamide; DNP, deoxyribose nucleoprotein; HAMA, Hamilton Anxiety Scale; HAMD, Hamilton Depression Scale; HC, healthy control; HCQ, hydroxychloroquine; MMSE, mini-mental state examination; SAS, Self-Rating Anxiety Scale; SLE, systemic lupus erythematosus; SLEDAI, systemic lupus erythematosus disease activity index.

### Brain regions exhibiting main effects of sex and diagnosis

**Figure 1:**
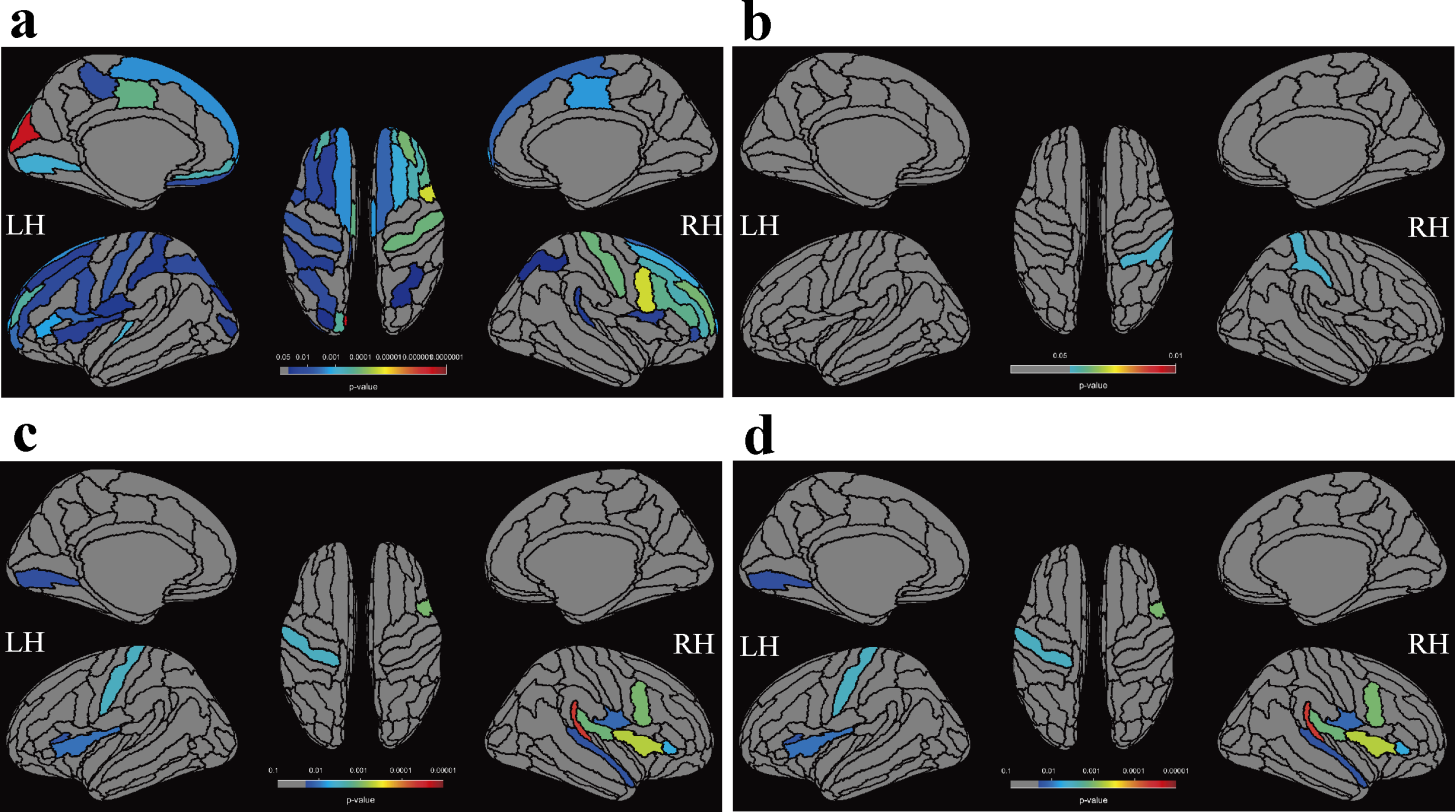
The main effect brain regions of the four groups of study participants based on whole-brain ROI. Note: (a) main effect brain regions of diagnosis on cortical thickness; (b) main effect brain regions of sex on cortical thickness; (c) main effect brain regions of diagnosis on sulcal depth; (d) and main effect brain regions of diagnosis on gyrification index. Holm-Bonferroni correction, p<0.05. The colour bar indicates the *P* value. LH, left hemisphere; RH, right hemisphere. ROI, regions of interest.

Significant diagnostic main effects of CT were observed in extensive brain regions including the superior frontal gyrus (G_front_sup), middle frontal gyrus (G_front_middle), inferior frontal sulcus (S_front_inf), middle-posterior part of the cingulate gyrus and sulcus (G_and_S_cingul-Mid-Post), subcentral gyrus and sulci (G_and_S_subcentral) and superior occipital gyrus (G_occipital_sup). A significant diagnosis main effect of CT was observed across these extensive brain regions, all with HC group>SLE group (table 2, figure 1a). A main effect of sex on CT was detected only in the right hemisphere’s Postcentral sulcus (S_postcentral), with the female group>male group (table 2, figure 1b). Diagnostic main effects for SD were observed in multiple regions including the central sulcus (S_central), superior segment of the circular sulcus of the insula (S_circular_insula_sup), vertical ramus of the anterior segment of the lateral sulcus (Lat_Fis-ant-Vertical) and lateral aspect of the superior temporal gyrus (G_temp_sup-Lateral), all showing HC group>SLE group (table 3, figure 1c). No sex main effect for SD was detected. A main effect of diagnosis for GI was observed in the left middle frontal sulcus (S_front_middle), right inferior segment of the circular sulcus of the insula (S_circular_insula_inf) and superior occipital gyrus (G_occipital_sup) regions and the HC group>SLE group in the left S_front_middle and right S_circular_insula_inf regions. The right G_occipital_sup region showed SLE group>HC group (table 3, figure 1d), with no observed main effect of sex on GI. No main effects were observed for FD.

**Table 2:** The main effect brain regions on CT of study participants

Main effect of diagnosis on CT	Brain regions	Grey matter thickness, mean (SD)		*F* value	Holm-Bonferroni corrected *P* value	Partial η²
		SLE	HC			
HC>SLE	G_cuneus_L	1.90 (0.10)	1.98 (0.09)	37.311155	0.000000	0.1527
	G_occipital_sup_L	2.09 (0.12)	2.17 (0.13)	22.230749	0.000244	0.0943
	G_and_S_transv_frontopol_L	2.43 (0.18)	2.54 (0.17)	20.029650	0.001421	0.0915
	G_and_S_cingul-Mid-Post_L	2.54 (0.13)	2.62 (0.14)	19.580268	0.000163	0.0820
	G_oc-temp_med-Lingual_L	1.94 (0.10)	2.00 (0.10)	19.271423	0.000904	0.0840
	G_front_sup_L	2.81 (0.17)	2.92 (0.14)	19.185426	0.001924	0.1135
	G_front_inf-Triangul_L	2.46 (0.17)	2.54 (0.15)	17.956778	0.001482	0.0594
	S_front_middle_L	2.33 (0.15)	2.42 (0.13)	17.846021	0.000261	0.0952
	G_front_inf-Opercular_L	2.58 (0.16)	2.67 (0.13)	17.529059	0.003901	0.0888
	S_suborbital_L	2.26 (0.21)	2.40 (0.18)	16.822298	0.000366	0.1167
	S_central_L	1.97 (0.15)	2.04 (0.11)	16.609832	0.006957	0.0672
	G_temp_sup-G_T_transv_L	2.16 (0.21)	2.24 (0.19)	16.509237	0.000780	0.0387
	G_and_S_frontomargin_L	2.27 (0.14)	2.36 (0.15)	16.329148	0.003483	0.0897
	S_precentral-inf-part_L	2.46 (0.17)	2.55 (0.14)	16.033311	0.010367	0.0786
	S_cingul-Marginalis_L	2.31 (0.14)	2.38 (0.13)	15.685706	0.009316	0.0639
	G_rectus_L	2.24 (0.14)	2.32 (0.15)	15.484149	0.012499	0.0720
	S_oc_middle_and_Lunatus_L	1.98 (0.13)	2.06 (0.14)	14.001131	0.035124	0.0825
	G_and_S_subcentral_L	2.48 (0.14)	2.56 (0.13)	13.829474	0.037450	0.0825
	S_front_sup_L	2.54 (0.16)	2.62 (0.12)	13.437308	0.029800	0.0757
	G_front_middle_L	2.53 (0.17)	2.63 (0.15)	13.061617	0.017774	0.0911
	S_intrapariet_and_P_trans_L	2.17 (0.11)	2.21 (0.12)	11.372149	0.015372	0.0296
	S_circular_insula_sup_L	2.67 (0.13)	2.72 (0.13)	10.955030	0.030184	0.0361
	S_oc_sup_and_transversal_L	2.13 (0.11)	2.17 (0.10)	10.443827	0.046459	0.0354
	S_postcentral_L	2.15 (0.13)	2.19 (0.12)	8.545305	0.034985	0.0252
	S_precentral-inf-part_R	2.47 (0.15)	2.56 (0.13)	25.450785	0.000024	0.0946
	S_front_inf_R	2.27 (0.14)	2.35 (0.12)	23.262776	0.000177	0.0873
	S_front_middle_R	2.33 (0.15)	2.41 (0.11)	22.041075	0.000107	0.0860
	G_front_middle_R	2.50 (0.16)	2.60 (0.13)	21.211856	0.000385	0.1074
	S_front_sup_R	2.56 (0.14)	2.65 (0.13)	20.452642	0.001088	0.1019
	G_and_S_transv_frontopol_R	2.43 (0.19)	2.54 (0.16)	20.034636	0.001575	0.0909
	G_and_S_cingul-Mid-Post_R	2.57 (0.14)	2.64 (0.12)	18.279286	0.001784	0.0682
	S_central_R	1.98 (0.13)	2.05 (0.11)	16.689615	0.000130	0.0794
	G_front_sup_R	2.83 (0.17)	2.93 (0.16)	15.501825	0.004285	0.0859
	S_orbital_lateral_R	2.23 (0.22)	2.34 (0.19)	14.097096	0.017787	0.0681
	S_intrapariet_and_P_trans_R	2.15 (0.11)	2.19 (0.11)	11.441521	0.048884	0.0324
	G_front_inf-Opercular_R	2.61 (0.15)	2.68 (0.15)	10.961160	0.027861	0.0526
	G_temp_sup-Plan_tempo_R	2.35 (0.20)	2.43 (0.17)	6.949980	0.036315	0.0455

Main effect of sex on CT	Brain regions	Grey matter thickness, mean (SD)		*F* value	Holm-Bonferroni corrected *P* value	
		Male	Female			
Female>male	S_postcentral_R	2.13 (0.13)	2.17 (0.11)	12.301435	0.043541	0.0271

CT, cortical thickness; HC, healthy control; L, left hemisphere; R, right hemisphere; SLE, systemic lupus erythematosus.

**Table 3:** The main effect brain regions on sulcus depth and gyrification index of study participants

Main effect of diagnosis on SD	Brain regions	Grey sulcal depth, mean (SD)		*F* value	Holm-Bonferroni corrected *P* value	Partial η²
		SLE	HC			
HC>SLE	S_central_L	13.39 (0.95)	13.85 (0.76)	14.921140	0.002924	0.0679
	S_circular_insula_sup_L	23.87 (1.45)	24.57 (1.51)	12.788980	0.010184	0.0538
	G_oc-temp_med-Lingual_L	8.57 (1.20)	9.18 (1.42)	12.180617	0.021032	0.0519
	Lat_Fis-ant-Vertical_L	13.36 (1.49)	14.02 (1.72)	8.789037	0.017104	0.0411
	G_temp_sup-Plan_tempo_R	8.44 (0.89)	9.12 (1.09)	26.402407	0.000044	0.1062
	S_circular_insula_sup_R	24.01 (1.29)	24.88 (1.53)	22.534938	0.000500	0.0877
	S_precentral-inf-part_R	12.15 (1.47)	12.92 (1.07)	20.366928	0.000998	0.0836
	Lat_Fis-post_R	25.63 (1.49)	26.30 (1.46)	15.730869	0.001139	0.0497
	Lat_Fis-ant-Horizont_R	13.53 (1.02)	14.14 (1.27)	14.132790	0.004993	0.0668
	G_temp_sup-Lateral_R	4.61 (0.65)	5.03 (1.05)	13.487362	0.017503	0.0556
	G_and_S_subcentral_R	10.28 (0.91)	10.64 (1.02)	6.578465	0.011098	0.0342

Main effect of diagnosis on GI	Brain regions	Grey gyrification index, mean (SD)		*F* value	Holm-Bonferroni corrected *P* value	
HC>SLE	S_front_middle_L	29.97 (1.69)	30.34 (1.25)	10.036755	0.008949	0.0153
	S_circular_insula_inf_R	23.03 (1.47)	23.80 (1.78)	14.387956	0.005603	0.0535
SLE>HC	G_occipital_sup_R	29.65 (1.44)	29.06 (1.31)	6.790359	0.039581	0.0451

GI, gyrification index; HC, healthy control; L, left hemisphere; R, right hemisphere; SD, sulcal depth; SLE, systemic lupus erythematosus.

### Brain regions exhibiting ‘sex × diagnosis’ interaction

**Figure 2:**
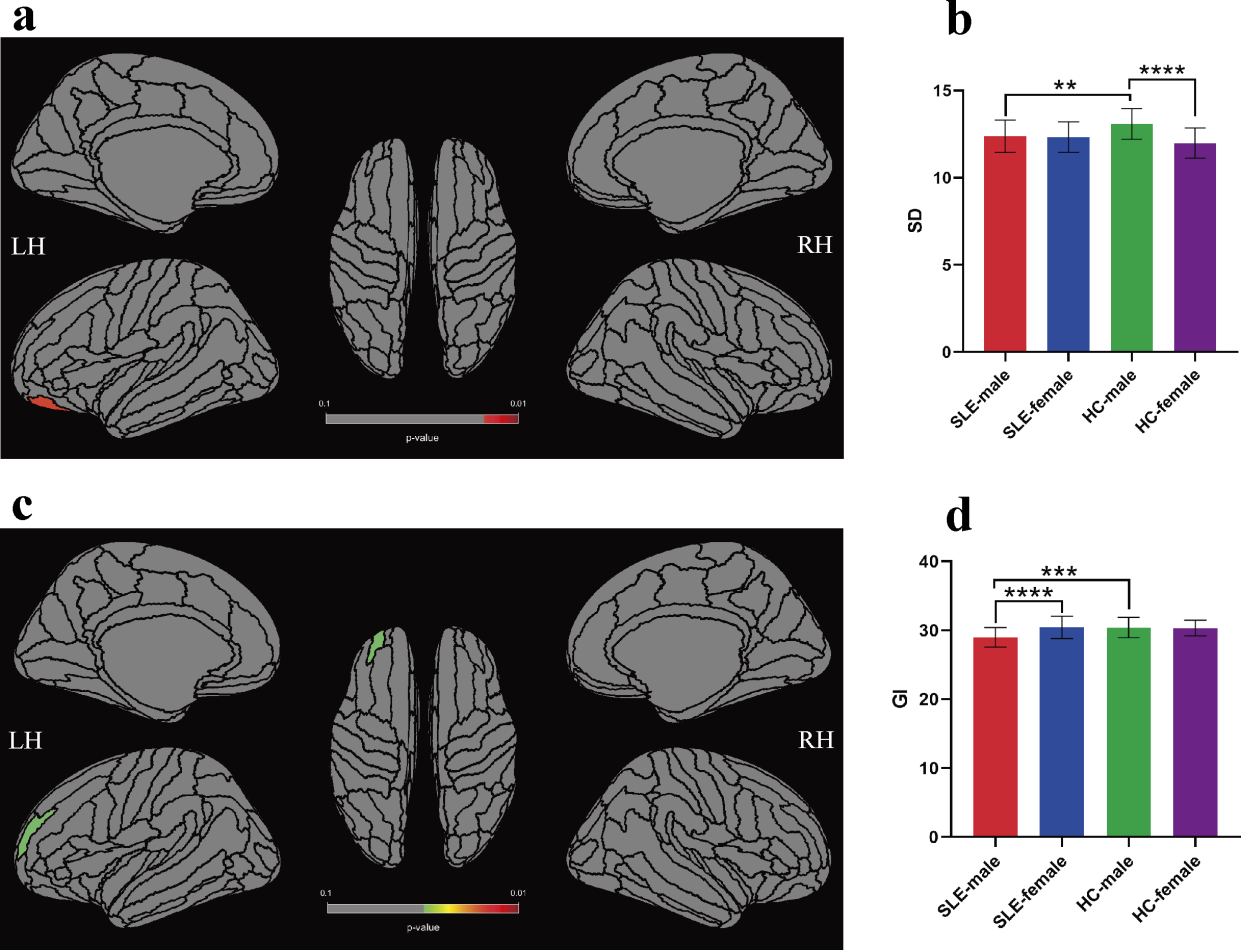
The inter effect of ‘sex×diagnosis’ brain regions of study participants based on whole-brain ROI. Note: (a) The interaction between sex and diagnosis on sulcal depth was observed in the left hemisphere S_orbital-H_Shaped, Holm-Bonferroni correction, *F* value=14.8458, *p* value=0.0150. (b) Comparison of sulcal depth values in brain regions for ‘sex×diagnosis’ interaction effects, with simple effect analysis results shown. (c) The interaction between sex and diagnosis on gyrification index was observed in the left hemisphere S_front_middle, Holm-Bonferroni correction, *F* value=14.1650, *p* value=0.0313. (d) Comparison of gyrification index values in brain regions for sex×diagnosis interaction effects, with simple effect analysis results shown. *p<0.05; **p<0.01; ***p<0.001; ****p<0.0001. The colour bars indicate *P* values. ROI, regions of interest; SLE, systemic lupus erythematosus.

A ‘sex×diagnosis’ interaction in SD was observed in the left orbital sulci (H-shaped) region (S_orbital-H_Shaped) (Holm-Bonferroni corrected, *F*=14.8458, p=0.0150, partial η² = 0.072). Simple effects analysis indicated: male HC group>Male SLE group (p=0.0039) and male HC group>female HC group (p<0.0001) (figure 2a,b). A ‘sex×diagnosis’ interaction in GI was observed in the left middle frontal sulcus (S_front_middle) region (Holm-Bonferroni corrected, *F*=14.1650, p=0.0313, η² = 0.069). Simple effect analysis indicated that the female SLE group>male SLE group (p<0.0001) and the male HC group>male SLE group (p=0.0003) (figure 2c,d). No ‘sex×diagnosis’ interaction effects were observed in CT or FD.

### Correlation analysis

In the male and female SLE groups, the mean values of SD, FD, GI and CT were extracted from the brain regions showing interaction effects and were used in conducted correlation analyses with SLEDAI score, HAMA, HAMD, MMSE, BDI and SAS scores respectively. Results revealed no significant correlations between structural indicators in interaction brain regions and either disease activity in SLE patients or scores on psychological assessment scales.

## Discussion

Sex main effects, diagnosis main effects and “sex×diagnosis’ interactions in CT and cortical complexity between SLE patients and HC were investigated in this study for the first time using ROI-based SBM analysis. The T1- and T2-weighted cranial MRI scans of all included SLE patients were normal, and none of them had a history of severe or overt neuropsychiatric disorders. However, patients with mild emotional and/or cognitive deficits identified by neuropsychological testing were not excluded. This research approach made it possible to look into early brain structural remodelling that might occur before the appearance of overt clinical symptoms of NPSLE, because patients with mild emotional or cognitive symptoms identified by these scales were not excluded. The duration of the disease, SLEDAI scores, psychological scale scores (HAMA, HAMD, MMSE, BDI, SAS) and the prevalence of positive antiphospholipid and anti-P0 antibodies were not different statistically significantly between male and female SLE patients (p>0.05). Furthermore, by controlling for age and years of education to minimise confounding factors, we can more reliably reveal the independent and interactive effects of sex and disease on brain structure.

SBM is an imaging method that can measure the cerebral cortex’s multidimensional morphological characteristics, enabling the accurate evaluation of cortical parameters. CT is defined as the geometric distance between the inner and outer surfaces of the cerebral grey matter.^14^ CT differs between diseases and across disease stages within the same condition,^15^ with CT abnormalities potentially affecting intrinsic brain connectivity in non-NPSLE patients.^16^ SD denotes the depth of cerebral surface sulci and is positively correlated with the network centrality of sulcal networks. Research indicates that deeper and earlier-developed sulci possess stronger long-range connectivity capabilities.^17^ FD serves as an indicator for evaluating the topological complexity of an object,^18^ reduced complexity in brain activity signifies diminished flexibility and efficacy in the integrative functions of the nervous system.^19^ GI quantifies the amount of cortex buried within gyral folds relative to the visible outer cortex.^20^ In healthy individuals, cortical GI positively correlates with intelligence,^21^ whereas in pathological states (such as childhood epilepsy), verbal IQ negatively correlates with GI.^22^ Current literature on SD, FD and GI in SLE remains limited. A comprehensive analysis of these metrics would provide a more holistic understanding of grey matter structural alterations in SLE patients.

### The impact of SLE disease state on brain structure

Cortical atrophy has been extensively documented in SLE patients. Current understanding suggests that SLE-associated brain damage involves multiple interrelated mechanisms, including disruption of the blood-brain barrier, tissue and nerve injury mediated by immune autoantibodies (such as anti-P0 antibodies, anti-U1-RNP antibodies, anti-ACL antibodies, etc) or pro-inflammatory factors (such as IL-1, IL-6, IL-8, TNF-α) mediated tissue and neural injury, as well as vascular obstruction caused by thrombosis, vasculitis and atherosclerotic changes;^23^,^25^ progressive diffuse neuronal loss ultimately leads to cortical atrophy. In this study, compared with the HC group, SLE patients exhibited extensive cortical thinning. Cortical thinning primarily affected bilateral sensorimotor cortex (pre/postcentral gyrus), frontal lobe (including superior, middle and inferior frontal gyri) and cingulate gyrus—core regions for sensorimotor processing, higher-order cognition and emotional integration. This finding aligns with previous studies reporting cortical thinning across multiple brain regions in neuropsychiatric SLE patients,^11 12 26^ with similar patterns observed even in non-neuropsychiatric SLE patients.^9 27^ This suggests widespread neuropathological changes in SLE, potentially explaining the high prevalence of neuropsychiatric symptoms (eg, headaches, seizures, cognitive impairment and mood disorders) in this population.^28^ As a key component of the limbic system, the posterior cingulate cortex plays a central role in memory, emotion and behavioural regulation.^29^ Epidemiological studies indicate significantly higher anxiety rates among SLE patients compared with HC.^30^ However, existing research on the association between anxiety symptoms and brain structure remains inconclusive, with some studies failing to identify significant correlations.^31 32^ Similarly, this study did not observe correlations between brain structure in interacting regions and anxiety scale scores. This may suggest that such structural alterations constitute a form of underlying neurobiological vulnerability rather than being the sole decisive cause directly leading to specific clinical symptoms. However, the absence of certain psychological assessment scales in this study means that the relationship between anxiety symptoms and brain structural alterations in SLE patients requires further confirmation. In this study, the observed shallowing of the SD was observed in brain regions associated with auditory processing, language and emotional regulation, including the central sulcus, temporal lobe, lateral sulcus and upper segment of the insular sulcus. Research has indicated that bilateral shallowing of the central sulcus connecting the precentral gyrus (motor cortex) and postcentral gyrus (sensory cortex) appears linked to deficits in social and physical pleasure in patients with major depressive disorder.^33^ The shallow central sulcus observed in SLE patients in this study may be associated with their underlying emotional disturbances and reduced social and physical activity, though this inference requires further validation through additional research. Furthermore, this study identified a diagnostic main effect of SLE disease status on GI in the left S_front_middle, right S_circular_insula_inf and G_occipital_sup regions. Specifically, GI values in the HC group were significantly higher than those in the SLE group in the S_front_middle and S_circular_insula_inf, while in the G_occipital_sup, GI values were significantly higher in the SLE group than in the HC group. Our team’s recent research also reported reduced GI in the left superior frontal gyrus of SLE patients compared with the HC group, with this reduction being more pronounced in SLE patients with comorbid anxiety and/or depression.^27^ GI has demonstrated heterogeneous changes in brain structures associated with other conditions such as Parkinson’s disease,^34^ major depressive disorder^35 36^ and schizophrenia.^37 38^ Collectively, these findings suggest that GI serves as a highly sensitive indicator of pathophysiological states, although exhibiting distinct response patterns across different brain functional regions. The precise underlying pathological mechanisms and comprehensive clinical implications require elucidation through meticulously designed longitudinal cohort studies.

### Impact of sex on brain structure

Previous studies have demonstrated that both the incidence and prevalence of SLE are higher in females than males.^39^ This may be attributed to sex hormones (such as oestrogen regulating T/B lymphocyte function), as well as abnormal X-chromosome inactivation, increased Toll-like receptor gene products and altered microRNA function.^40 41^ However, male SLE patients typically exhibit higher disease activity, with more pronounced rates of organ damage, such as kidney and cardiovascular damage, as well as higher mortality,^5 6^ suggesting sex factors may significantly influence SLE’s pathological progression and prognosis. In this study, a significant main effect of sex was observed in the right S_postcentral_R region, manifested as a slightly lower CT in males than females, independent of disease status. This indicates that sex differences in this brain region exist in both healthy individuals and SLE patients. This area is responsible for integrating somatosensory processing and risk perception.^42^ This sex difference, independent of disease status, reflects a baseline structural dimorphism; however, because no significant sex-by-diagnosis interaction was detected for CT in this region, we cannot infer disproportionate disease-related damage in male SLE patients relative to female SLE patients. This finding appears superficially contradictory to previous reports indicating more significant global GMV reduction in female SLE patients,^10^ yet it may profoundly reflect potential differences in brain injury patterns between the global level and local microstructural levels. Regarding healthy populations, multiple prior studies have identified greater primary CT in females,^43 44^ consistent with the present findings and further supporting biological sex differences in central nervous system structure. This study did not identify a sex main effect on SD, FD or GI. This may be because CT serves as a sensitive marker reflecting acute, focal grey matter damage associated with SLE, whereas SD, FD and GI may require more severe or persistent cumulative damage to reveal differences. Furthermore, all patients enrolled in this study were non-NPSLE cases who had no severe or overt neuropsychiatric manifestations, and their overall neurological damage severity may have remained at a relatively early stage. Consequently, the study failed to highlight the pervasive influence of sex factors on these specific indicators.

### Interaction between SLE disease state and sex on brain structure

This study identified a significant ‘sex×diagnosis’ interaction effect for SD in the left cerebral hemisphere orbital sulci (H-shaped) region. However, neither a main effect of diagnosis nor a main effect of sex was observed for SD in this specific brain area. This clearly demonstrates that, within this particular functional brain region, the synergistic effect of sex and disease far outweighs their respective independent influences. Simple effects analysis further revealed that SD in male HC was higher than in female HC and also higher than in male SLE patients, suggesting that in healthy male brains, this region naturally possesses deeper sulcal structures. However, this structural advantage present in physiological conditions is markedly attenuated in male patients who unfortunately develop SLE. Previous studies have also documented widespread reduction in cortical SD among SLE patients compared with HC.^27^ This ‘H-shaped’ orbital sulcus partitions the ventral surface of the frontal lobe into four orbital gyri: the anterior orbital gyrus, posterior orbital gyrus, lateral orbital gyrus and medial orbital gyrus.^13^ The lateral orbital gyrus constitutes part of the orbitofrontal cortex, a critical subregion of the prefrontal cortex playing a key role in emotional regulation, decision-making and social behaviour.^45 46^ Previous studies have demonstrated abnormalities in prefrontal-limbic perfusion kinetics and functional connectivity in NPSLE patients presenting with severe anxiety and depressive symptoms.^47^ Although this region has been linked to emotional processing and antidepressant response in other clinical contexts,^48 49^ we caution that our cross-sectional structural data do not permit direct inferences about decision-making, social behaviour or treatment targets in this SLE cohort. Any such functional or therapeutic implications remain speculative and require longitudinal or multimodal validation. This study also observed a significant ‘sex×diagnosis’ interaction for GI in the left S_front_middle. Simple effects analysis indicated that GI values in the male SLE group were significantly lower than those in the HC group; concurrently, GI values in the male SLE group were also significantly lower than those in the female SLE group. Notably, a main effect of diagnosis on GI was simultaneously observed in this brain region (SLE group significantly lower than HC group). This finding further confirms that both the SLE disease state itself and its interaction with sex significantly influence morphological characteristics in this region. Notably, the detected interaction effects yielded small-to-medium effect sizes (partial η² = 0.072 for sulcal depth in left S_orbital-H_Shaped and partial η² = 0.069 for GI in left S_front_middle). However, these estimates should be interpreted with caution given the modest male SLE sample size (n=30) and unequal group distribution, which can inflate the SE of interaction terms and increase susceptibility to outliers in the smaller subgroup. The middle frontal sulcus lies within the middle frontal gyrus and exhibits a discontinuous configuration.^13^ The middle frontal gyrus serves as the core of the prefrontal network, integrating cognitive, motor, emotional and social behaviours. In this study, a significant main effect of CT was observed in both the medial frontal gyrus and medial frontal sulcus regions of the bilateral cerebral hemispheres, with HC group CT values significantly higher than those in the SLE group.

Because the sex main effect on CT was restricted to the right postcentral sulcus and no significant correlations with clinical or psychological scales were found in interaction-significant regions, we cannot infer disproportionately greater male damage here or direct functional consequences. Although altered functional connectivity in the middle frontal gyrus has been reported in NPSLE,^50^ direct extrapolation to SLE patients without severe neuropsychiatric manifestations is unwarranted. To date, no research has reported on the interactive effects of SLE disease state and sex differences on GI. Future studies should integrate SLE-specific pathological mechanisms to explore the patterns of GI changes in this disease and their clinical significance.

### Potential biological mechanisms underlying ‘sex×diagnosis’ interactions

The observed sex×diagnosis interaction likely results from the convergence of sex-specific neurobiological endowments and SLE-specific pathophysiology. We propose three interrelated mechanisms.

First, sex hormone–immune crosstalk may play a central role. In healthy males, testosterone promotes cortical maturation and supports deeper sulcal morphology.^51^ In SLE, however, male patients often exhibit lower testosterone levels and elevated oestradiol-to-testosterone ratios.^52^ This dual hormonal perturbation simultaneously reduces androgen-mediated neuroprotection and exacerbates pro-inflammatory cytokine cascades, potentially explaining why male SLE patients lose their baseline sulcal depth advantage in the orbitofrontal cortex and show markedly reduced gyrification in the middle frontal sulcus.

Second, sex-differential autoantibody and microvascular profiles may mediate region-specific damage. In our cohort, male SLE patients showed significantly higher positivity for anti-Sm and anti-U1RNP antibodies—both implicated in endothelial activation and microvascular injury. Coupled with the established male predominance in cardiovascular and renal involvement in SLE,^5^ these autoantibody and vasculopathic burdens may preferentially compromise the highly vascularised orbitofrontal and middle frontal regions, accelerating microstructural rarefaction in male patients.

Third, sex-dimorphic neurodevelopmental trajectories may establish differential resilience. Males typically exhibit greater sulcal depth and distinct gyrification in prefrontal regions, a pattern reflecting foetal testosterone-driven connectivity optimisation. Chronic inflammation in SLE—mediated by cytokines such as IL-1β, IL-6 and TNF-α—may disrupt this male-typical maturational programme, effectively ‘flattening’ cortical folding patterns.^53^ The disproportionate reduction of gyrification in the left middle frontal sulcus, a core hub for executive control, supports this developmental vulnerability hypothesis.

Together, these mechanisms suggest that the sex×diagnosis interaction is biologically grounded in hormonally modulated immunity and sex-dimorphic neurodevelopment, rather than representing a statistical artefact. Future studies incorporating longitudinal hormonal profiling, detailed autoantibody characterisation and blood–brain barrier permeability imaging are warranted to disentangle these pathways.

Several limitations should be acknowledged. First, the relatively small sample size of male SLE patients (n=30) and the resultant unequal distribution across the four groups may limit statistical power and affect the stability of the sex-by-diagnosis interaction estimates. Unequal group sizes can inflate the SE of interaction terms and increase susceptibility to outliers in the smaller subgroup. Future multicentre studies with larger and more balanced male cohorts are warranted to confirm the stability of these interaction effects. Second, the cross-sectional design precludes causal inferences regarding the temporal dynamics of cortical alterations. Longitudinal investigations are needed to clarify whether these structural changes represent reversible or progressive neuropathological processes. Third, the two-way ANOVA was adjusted only for age and years of education as covariates. Although these are established confounders in cortical morphometry, we acknowledge that other clinical factors—including disease duration, disease activity, organ involvement, cumulative glucocorticoid exposure and immunosuppressive medication use—may also influence brain structure. Given the modest male SLE sample size, incorporating multiple additional covariates simultaneously would risk model overfitting and substantially reduce statistical power. Future multicentre studies with larger cohorts should validate these findings while controlling for these important clinical variables. Fourth, the definition of ‘normal MRI’ in this study was based on conventional T1- and T2-weighted sequences without gross structural abnormalities. Microinfarcts, white matter hyperintensities or cerebral microbleeds were not systematically excluded because advanced sequences (eg, FLAIR, SWI, or high-resolution structural imaging) were not acquired. Future studies should incorporate these sequences to rule out subtle cerebrovascular pathology.

## Conclusion

In summary, this study found that compared with the HC group, patients with SLE exhibited widespread CT reduction and SD shallowing, with a sex main effect on CT observed only in the right postcentral sulcus (female>male). More importantly, our study provides the first observation of a ‘sex×diagnosis’ interaction affecting cortical complexity. These MRI-based imaging findings highlight the diverse patterns of brain structural alterations in SLE patients and may generate hypotheses regarding the regional specificity and sex differences of SLE-associated neuropathological changes.

## Data Availability

Data are available upon reasonable request.
